# Correlation of Severity of Human Tick-Borne Encephalitis Virus Disease and Pathogenicity in Mice

**DOI:** 10.3201/eid2409.171825

**Published:** 2018-09

**Authors:** Chaitanya Kurhade, Sarah Schreier, Yi-Ping Lee, Loreen Zegenhagen, Marika Hjertqvist, Gerhard Dobler, Andrea Kröger, Anna K. Överby

**Affiliations:** Umeå University, Umeå, Sweden (C. Kurhade, Y.-P. Lee, A.K. Överby);; Helmholtz Centre for Infection Research, Braunschweig, Germany (S. Schreier, L. Zegenhagen, A. Kröger);; Otto-von-Guericke-University Magdeburg, Magdeburg, Germany (S. Schreier, A. Kröger);; Public Health Agency of Sweden, Solna, Sweden (M. Hjertqvist);; Bundeswehr Institute of Microbiology, Munich, Germany (G. Dobler);; DZIF Partner Site Munich, Munich (G. Dobler)

**Keywords:** Tick-borne encephalitis virus, flavivirus, tick-borne encephalitis, pathogenesis, vector-borne infections, arboviral diseases, Sweden, Germany, ticks, viruses

## Abstract

We compared 2 tick-borne encephalitis virus strains isolated from 2 different foci that cause different symptoms in tick-borne encephalitis patients, from neurologic to mild gastrointestinal symptoms. We compared neuroinvasiveness, neurovirulence, and proinflammatory cytokine response in mice and found unique differences that contribute to our understanding of pathogenesis.

Tick-borne encephalitis (TBE) is an emerging arthropod-borne viral (arboviral) disease in Europe and Asia characterized by severe central nervous system (CNS) disease in humans. New areas of endemicity and increased TBE incidence have been reported (*1*). In Sweden, TBE cases have increased dramatically; during 2017, a record year, 391 cases were reported, compared with 238 cases during 2016. TBE virus (TBEV) is transmitted by tick bites and ingestion of contaminated milk (*2*,*3*). Infection (TBEV, European subtype) usually follows a biphasic course in which, during the primary phase, patients show symptoms of fatigue, headache, myalgia, and fever, followed by a second phase of neurologic involvement with signs of meningitis, encephalitis, and paralysis and high fever. Neurologic sequelae develop in 20%–60% of cases (*4*–*7*). The ability of the virus to cause CNS disease (neurovirulence) depends on its ability to enter the brain (neuroinvasiveness).

Recently, a focus of TBE in southeastern Germany was identified with 5 patients (2005–2011), who showed only mild gastrointestinal and constitutional symptoms, without neurologic symptoms. One strain, MucAr HB171/11, was isolated from 6 questing adult *Ixodes ricinus* ticks from this natural focus (49°17′N, 12°12′E) (*8*). To investigate this low pathogenic strain and the absence of neurologic symptoms, we compared its pathogenesis with another European strain, Torö-2003. Torö-2003 was rescued from a cDNA infectious clone (*9*) generated from RNA extracted from a pool of *I. ricinus* ticks (9 adults, 106 nymphs) collected in September 2003 on the island of Torö (58°49′N, 17°50′E) in the Stockholm archipelago of Sweden (*10*). In the focus on Torö, 32 TBE patients (1986–2016) were reported. Data on 4 of these TBE case-patients show relatively mild neurologic disease with a few days of hospitalization for 2 of them. In the mouse model, Torö-2003 shows similar pathogenicity as the highly virulent Hypr strain (*9*). Knowledge of differential clinical courses and severity of disease caused by strains of TBEV can be an important criterion for diagnosing and treating the disease.

## The Study

All animal experiments were performed in compliance with the German animal welfare law (TierSchG BGBl. S. 1105; 25.05.1998). Mice were housed and handled in accordance with good animal practice as defined by the Federation for Laboratory Animal Science Associations. All animal experiments were approved by the responsible state office (Lower Saxony State Office of Consumer Protection and Food Safety) under permit no. AZ 33.9-42502-04-11/0528. Experiments were performed in the Biosafety Level 3 facility at the Helmholtz Center for Infection Research (Braunschweig, Germany). Mice used for primary cell isolation were maintained under specific pathogen-free conditions, and studies were conducted according to the guidelines set out by the Regional Animal Ethical Committee (Umeå, Sweden; approval no. A77-14).

**Figure 1:**
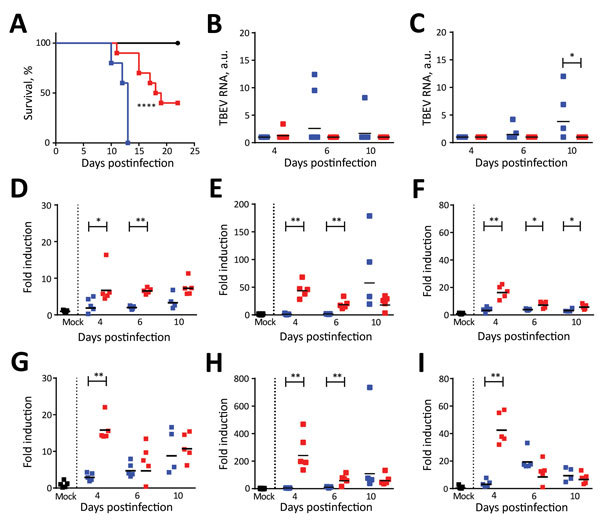
Survival analysis and TBEV burden in peripheral organs of Torö-2003–infected and HB171/11-infected C57BL/6 mice. A) Survival analysis of ten 6–10-week-old female C57BL/6 mice after subcutaneous inoculation with phosphate-buffered saline (mock, black) or with 10^4^ focus forming units (FFU) of Torö-2003 (blue) or HB171/11 (red) in 100 μL phosphate-buffered saline. Survival differences were tested for statistical significance by log-rank test. B, C) Viral burdens in spleen (B) and lymph node (C) after subcutaneous infection of Torö-2003 or HB171/11 (10^4^ FFU, n = 5) were measured by quantitative PCR and normalized to intracellular glyceraldehyde 3-phosphate dehydrogenase (GAPDH) levels as previously described (*14*). Each data point represents an individual mouse. D–I) Cytokine response in spleen (D–F) and lymph node (G–I). Expression levels of GAPDH, TNF-α (D,G), IL-6 (E,H), and CXCL-10 (F,I) were determined by validated QuantiTect primer assays (QIAGEN, Hilden, Germany) and quantitative PCR from organs prepared in B and C. Signals of indicated mRNA were normalized to the GAPDH mRNA signal. Bars indicate mean values. Asterisks indicate statistical significance calculated by Mann-Whitney test (*p<0.05; **p<0.01). Horizontal black bars indicate mean values. a.u., arbitrary units; CXCL, CXC motif ligand; IL, interleukin; TBEV, tick-borne encephalitis virus; TNF, tumor necrosis factor.

To assess the pathogenicity of the Torö-2003 and HB171/11 strains, we challenged C57BL/6 mice with 10^4^ focus-forming units of Torö-2003 and HB171/11 (second passage in Vero cells) subcutaneously. Mice were highly susceptible to Torö-2003 infection and 100% succumbed to the infection; median survival time was 13 days. Mice showed paralysis, lethargy, hunchback posture, fur ruffling, and weight loss 2 days before death. In contrast, only 60% of the HB171/11-infected mice died (median survival time 18.5 days) (Figure 1, panel A). Analysis of viral RNA in peripheral organs after infection showed viral replication of Torö-2003 6 days postinfection (dpi) in the spleen (Figure 1, panel B). In inguinal lymph nodes (Figure 1, panel C) and lung (data not shown), we detected Torö-2003 RNA only at 10 dpi. In contrast, viral RNA from HB171/11 could hardly be detected in peripheral organs. Because gastrointestinal and constitutional symptoms reported for HB171/11 in humans (*8*) are hardly detectable in mice, we analyzed viral RNA in colon, appendix, and small intestine. No viral RNA was detected in these tissues (data not shown).

To investigate whether infection with the different virus strains changes immune response, we looked for proinflammatory gene induction in lymphoid tissue. Tumor necrosis factor–α, interleukin-6, and CXC motif ligand–10 were highly up-regulated in spleen and lymph nodes on infection with HB171/11, compared with Torö-2003 4 dpi. At later time points, we detected similar expression levels in Torö-2003–infected and HB171/11-infected mice (Figure 1, panels D–I). Macrophages and monocytes are potential producers of these cytokines and may influence immune response to control virus replication of HB171/11 in the periphery.

**Figure 2:**
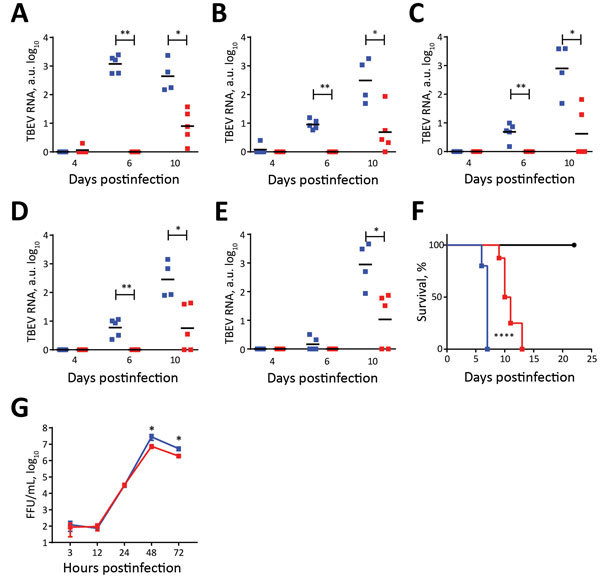
TBEV burden in central nervous system (CNS) of mice. A–E) Five 6–10-week-old female C57BL/6 mice were infected subcutaneously with 104 FFU of Torö-2003 (blue) or HB171/11 (red), and viral burden in CNS tissue (olfactory bulb [A], cerebrum [B], cerebellum [C], brain stem [D], and spinal cord [E]) was measured by quantitative PCR and normalized to intracellular glyceraldehyde 3-phosphate dehydrogenase levels. Horizontal black bars indicate mean values. Statistical significance calculated by the Mann-Whitney test (*p<0.05; **p<0.005). F) Survival analysis of C57BL/6 mice after intracranial inoculation with phosphate buffered saline (mock, black) or with 100 FFU of Torö-2003 (n = 8) or HB171/11 (n = 10) in 20 μL phosphate buffered saline. For intracranial infections, mice were anesthetized by intraperitoneal injection with a mixture of ketamine (100 μg/g body weight) and xylazine (5 μg/g body weight). Survival differences were tested for statistical significance by log-rank test (****p<0.0005). G) Viral replication kinetics in primary cortical neurons. Primary cortical neurons were isolated as described previously (*11*). The neurons were infected with Torö-2003 or HB171/11 strain with 0.001 multiplicity of infection at day 7 postseeding, and viral growth was determined at indicated time points by focus forming assay, as previously described (*15*). Statistical significance was calculated using unpaired t test (*p<0.05). a.u., arbitrary units; FFU, focus-forming units; TBEV, tick-borne encephalitis virus.

Because HB171/11 caused only mild gastrointestinal and constitutional symptoms without specific neurologic symptoms, we hypothesized that HB171/11 might be less neuroinvasive than Torö-2003. To assess this hypothesis, we infected mice subcutaneously and isolated different parts of the CNS (olfactory bulb, cerebrum, cerebellum, brain stem, and spinal cord) at different times after infection and analyzed viral load in the CNS. At 6 dpi, we detected Torö-2003 virus in almost all the CNS regions; replication was highest in the olfactory bulb. At 10 dpi, Torö-2003 virus replication further increased in all parts except the olfactory bulb, where viral burden was maintained. For HB171/11, we detected no viral RNA at 6 dpi and only low levels of virus in most CNS parts at 10 dpi (Figure 2, panels A–E), indicating delayed neuroinvasiveness of HB171/11. 

Next, we investigated the neurovirulence of the different strains. We injected mice with 100 focus-forming units of virus directly into cerebral cortex through the intracranial route. Torö-2003 was highly pathogenic, leading to 100% deaths; median survival was 7 days. We observed lower neurovirulence for HB171/11, and survival was a median of 10.5 days. 

To explore the relationship between mice pathogenicity and viral replication in target cells, we analyzed replication in neurons. We isolated primary cortical neurons from C57BL/6 mice infected with 0.001 multiplicity of infection and measured progeny particles by focus-forming assay (*11*). Both strains replicated to the same level at early time points; later in infection (48 and 72 h), Torö-2003 replicated to higher levels compared with HB171/11 (Figure 2, panel G).

Taken together, HB171/11 shows lower neurovirulence in mice, probably because of reduced replication in neurons. However, we cannot exclude that other cell types within the CNS (astrocytes and microglia) also could contribute to the lower neurovirulence. The low neurovirulence in combination with the slower neuroinvasiveness, resulting from low replication in the periphery and high up-regulation of proinflammatory cytokines, make the HB171/11 less pathogenic in the mouse model.

## Conclusions

TBEV is spreading into new regions in Europe: Sweden, Norway, Finland, France, the Netherlands, Italy, and Switzerland (*1*). The typical symptoms of infection are meningitis, encephalitis, and paralysis. However, recent reports also indicate gastrointestinal problems (*8*). Such new strains that cause these rare symptoms complicate the diagnosis of TBEV infection and raise the question of the number of unrecognized TBE cases. We characterized the pathogenesis and immune response of 2 European isolates of TBEV from infection foci that coincide with human cases displaying completely different disease symptoms. To characterize these clinical manifestations of disease, we used C57BL/6 mice to study TBEV pathogenesis. These mice are susceptible to infection and develop encephalitis without the need for adaptation of the virus isolates. The pathogenicity of the 2 virus strains clearly differed. We could not detect gastrointestinal symptoms in the HB171/11-infected mice, but the low-virulence phenotype of HB171/11 could be mimicked. The mice showed a strong cytokine response in periphery, low and delayed neuroinvasiveness, and low neurovirulence that, when translated into humans, might explain the lack of neurologic symptoms. Because genetic changes in TBEV could affect pathogenicity (*12*,*13*), future molecular studies are needed to determine the low pathogenicity of and enhanced immune response against low-virulence strain HB171/11.
